# India's achievement towards sustainable Development Goal 6 (Ensure availability and sustainable management of water and sanitation for all) in the 2030 Agenda

**DOI:** 10.1186/s12889-022-14316-0

**Published:** 2022-11-21

**Authors:** Sourav Biswas, Biswajit Dandapat, Asraful Alam, Lakshminarayan Satpati

**Affiliations:** 1 Department of Population & Development, International Institute for Population Sciences, Govandi Station Road, Opposite Sanjona Chamber, Deonar, Mumbai, Maharashtra 400088 India; 2 Department of Geography, Ravenshaw University, College Road, Cuttack, Odisha 753003 India; 3 Department of Geography, Serampore Girls’ College, University of Calcutta, Serampore, 712201 India; 4 Professor of Geography & Director, UGC-HRDC, University of Calcutta, Kolkata, 700019 India

**Keywords:** Sustainable Development, SDG 6, Public Health, Clean Water and Sanitation, Universal coverage of latrine and sanitation

## Abstract

**Background:**

Clean water and sanitation are global public health issues. Safe drinking water and sanitation are essential, especially for children, to prevent acute and chronic illness death and sustain a healthy life. The UN General Assembly announced the 17 Sustainable Development Goals (SDGs) and 169 targets for the 2030 Agenda on 25 September 2015. SDG 6 is very important because it affects other SDG (1, 2,3,5,11,14 and 15). The present study deals with the national and state-wise analysis of the current status and to access deficiency of India's achievement towards SDG 6 (clean water and sanitation for all) for the 2030 agenda based on targets 6.1, 6.2,6.4,6.6 from 2012 to 2020.

**Materials and methods:**

Data of different indicators of SDG 6 are collected from different secondary sources—NSS 69th (2012) and 76th (2018) round; CGWB annual report 2016–2017 and 2018-2019; NARSS (2019–2020); SBM-Grameen (2020). To understand overall achievement towards SDG 6 in the 2030 agenda, the goal score (arithmetic mean of normalised value) has been calculated.

**Major findings:**

According to NSS data, 88.7% of Indian households had enough drinking water from primary drinking water sources throughout the year, while 79.8% of households had access to toilet facilities in 2018. As per the 2019–2021 goal score for States and UTs in rural India based on SDG 6 indicator, SDG 6 achiever States and UTs (100%) are Sikkim, Himachal Pradesh, Andaman and Nicobar Islands.

**Conclusion:**

Drinking water and sanitation for all ensure a healthy life. It is a matter of concern for the government, policymakers, and people to improve the condition where the goal score and indicator value of SDG 6 are low.

## Background

Clean Water and sanitation are global public health issues. "Water collected from sources like—piped water into dwelling, piped water into yard/plot, household connection, public standpipes/tap, boreholes/tube well, protected dug wells, protected springs and rainwater collection and bottled water are considered as improved sources of drinking water. Drinking water collected from improved sources located on-premises, available when needed and free from faecal and contamination is known as safely managed drinking water" [1]. "Hygiene refers to conditions and practices that help maintain health and prevent the spread of diseases” [2]. Water, sanitation and hygiene are known as WASH. WASH includes the use of safe drinking water; safe disposal and management of human faecal matter, human waste (solid and liquid). Open defecation is much more common in rural India than in urban India. About 70% of the Indian population lives in rural areas. In fact, 89% of households without toilets were in rural areas, according to the 2011 census. Although the Indian government has spent decades building latrines and the country has had consistent economic progress, rural open defecation statistics have remained stubbornly high [3].Control of vector-borne diseases, handwashing practices. Open Defecation Free (ODF) is the termination of faecal-oral transmission in an open space or ending open defecation using a toilet. India has progressed in access to safe drinking water (tap/hand-pump/tube well) in the household from 38% in 1981 to 85.5% in 2011. Water, sanitation, and hygiene-related diseases are Infectious Diarrhoea, Typhoid and paratyphoid fevers, Acute hepatitis A, Acute hepatitis E and future F, Fluorosis, Arsenosis, Legionellosis, Methamoglobinamia, Schistosomiasis, Trachomaa, Ascariasis, Trichuriasis, Hookworm, Dracunculiasis, Scabies, Dengue, Filariasis, Malaria, Japanese encephalitis, Leishmaniasis, Onchocerciasisa, Yellow fever, Impetigo and Drowning [4]. The United Nations General Assembly declared 2008 the International Year of Sanitation to recognise the critical need for increased political awareness and action on sanitation. The purpose is to promote awareness and speed up progress toward the Millennium Development Goal of decreasing the proportion of people without access to basic sanitation by 2015. Due to poor sanitation, people suffer from bad health, lost income, inconvenience, and indignity. Despite this, billions of people worldwide do not have access to basic sanitation [4, 5]. According to WHO (2015), 2.4 billion people lack sanitation facilities, and 663 million people still lack access to safe and clean drinking water facilities [6]. WHO (2019) state that 3.3% of global death and 4.6% of DALYs is attributed to inadequate water, sanitation and hygiene condition. "Unsafe sanitation is responsible for 775,000 deaths per year, 5% death in low-income countries due to unsafe sanitation, 15% of the world still practising open defecation [7]. "Age-standardized death rate attributable to unsafe water, sanitation, and hygiene (WaSH) (per 100,000 population) 268.587 in 1990, 239.719 in 1995, 210.642 in 2000, 180.757 in 2005, 143.453 in 2010 and 104.202 in 2016″ [7]. So safe drinking water and sanitation are essential, especially for children, to prevent acute and chronic illness death and sustain a healthy life. After the Millennium Development goal, on 25 September 2015, in UN general assembly 17th sustainable development goal (SDG) and 169 targets set up for 2030 agenda [8, 9]. "SDG 6 is essential because it affects other SDG (1 – poverty eradication, 2 – ending hunger, 3 – healthy life and well–being, 4 – quality education, 5 – gender equality, 11 – inclusive cities, 14 – life below water and 15 – terrestrial ecosystem)" [10]. The present study deals with the national and state-wise analysis of current status and to access deficiency of India's Achievement towards SDG 6 (clean water and sanitation for all) for the 2030 agenda based on targets 6.1, 6.2, 6.4, 6.6 from 2012 to 2020. In this study, special focus is given to rural India.

Census of India continuously collecting data about drinking water and sanitation from all households in house listing and housing. “The National Statistical Office (NSO) Ministry of Statistics and Programme Implementation” (MOSPI), Government of India has been collecting data on housing condition, drinking water, sanitation and hygiene; those were collected by NSO from NSS 7th round (October 1953—March 1954) to NSS 23rd round (July 1968—June 1969), 28th round (October 1973—June 1974), 44th round (July 1988—June 1989), 49th round (January—June 1993), 54th round (January—June 1998) 58th round (July—December 2002), 65th round (July 2008—June 2009), 69th round (July—December 2012), and latest NSS 76th round. The Indian government has undertaken attempts to enhance drinking water and sanitation.

- 1949: The Environment Hygiene Committee advises that a clean water supply be provided to 90% of India's population within a 40-year timeframe.
- 1969: The National Rural Drinking Water Supply Program was initiated with UNICEF's technical assistance, and Rs.254.90 crore is spent on 1.2 million bore wells and 17,000 piped water supply systems during this phase.
- In 1972–73, the Government of India launched the Accelerated Rural Water Supply Programme (ARWSP) to assist states and union territories in expanding drinking water supply coverage.
- 1986: The National Drinking Water Mission (NDWM) was established. The National Drinking Water Mission was renamed the Rajiv Gandhi National Drinking Water Mission in 1991 (RGNDWM). The 73rd Constitutional Amendment mandates the provision of drinking water by Panchayati Raj institutions (PRIs).
- In 1986, the Central Rural Sanitation Programme (CRSP) was established to provide safe sanitation in rural regions. The Total Sanitation Campaign (TSC) was launched in 1999 to promote local sanitary marts and various technical choices to develop supply-led sanitation.
- 1999: The Total Sanitation Campaign (TSC) was launched in 1999 as part of the reform principles to provide sanitation facilities in rural regions to eliminate open defecation. Swajal Dhara, a national scale-up of sector reform, was launched in 2002. All drinking water programmers were placed under the RGNDWM's umbrella in 2004.
- 2005: The Indian government begins the Bharat Nirman Programme, aiming to improve housing, roads, power, telephone, irrigation, and drinking water infrastructure in rural regions [11].
- In 2009, the ARWSP was renamed the National Rural Drinking Water Programme (NRDWP). One of the goals was to allow all households, to the extent practicable, to have access to and utilise safe and adequate drinking water inside the premises.
- In 2012, The Nirmal Bharat Abhiyan was reformed and renamed (rural sanitation).
- The Swachh Bharat Mission was launched across the country on 2 October 2014 to achieve the objective of a clean India by 2 October 2019. (PM India).
- The current National Rural Drinking Water Programme (NRDWP) was reformed and incorporated under Jal Jeevan Mission (JJM) on 15 August 2019 to provide Functional Household Tap Connection (FHTC) to every rural household, i.e. Har Ghar Nal Se Jal (HGNSJ) by 2024. Jal Jeevan Mission (JJM) is a non-profit organisation.

The goals of SBM(Gmain) are to enhance the general quality of life in rural areas by fostering cleanliness, hygiene, and the elimination of open defecation. The Individual Household Latrines (IHHL) unit cost was increased from Rs. 10,000 to Rs. 12,000 rupees to accommodate for water availability. To meet the Swachh Bharat aim, improve rural sanitation coverage by 2 October 2019. Raising awareness and providing health education encourages communities and Panchayati Raj institutions to adopt sustainable sanitation practices and infrastructure. Encourage the use of cost-effective and suitable sanitation methods that are environmentally safe and long-lasting. Develop community-managed sanitation systems in rural regions, concentrating on scientific Solid and Liquid Waste Management systems for overall cleanliness [11, 12].

In New York in 2000, 189 nations approved the Millennium Declaration for 2015, promising to work together to create a safer, more prosperous, and equal world. There are eight objectives, seven of which deal with sanitation and hygiene (target 7. C – Reduce the share of the population without sustainable access to clean drinking water and basic sanitation by 2015). (Millennium Development Goal of the United Nations) Following the millennium development goal (SDG), the United Nations General Assembly approved 17 sustainable development goals and 169 targets for the 2030 Agenda for Sustainable Development on 25 September 2015. Out of 17 SDGs, SDG 6 ensures availability and sustainable water and sanitation management. SDG 6 has different target for the year 2030—6.1: Achieve universal and equitable access to safe and affordable drinking water for all; 6.2: Achieve access to adequate and equitable sanitation and hygiene for all and end open defecation, paying particular attention to the needs of women and girls and those in vulnerable situations; 6.3: Improve water quality by reducing pollution, eliminating dumping and minimizing release of hazardous chemicals and materials, halving the proportion of untreated wastewater and substantially increasing recycling and safe reuse globally; 6.4: By 2030, substantially increase water-use efficiency across all sectors and ensure sustainable withdrawals and supply of freshwater to address water scarcity and substantially reduce the number of people suffering from water scarcity; 6.5: Implement integrated water resources management at all levels, including through transboundary cooperation as appropriate; 6.6: Protect and restore water-related ecosystems, including mountains, forests, wetlands, rivers, aquifers and lakes; 6.a: Expand international cooperation and capacitybuilding support to developing countries in water- and sanitation-related activities and programmes, including water harvesting, desalination, water efficiency, wastewater treatment, recycling and reuse technologies; 6.b: Support and strengthen the participation of local communities in improving water and sanitation management [8].

As the nodal institution for SDGs, NITI Aayog, the Government of India has striven to provide the necessary encouragement and support to forge collaborative momentum among them. Since 2018, the SDG India Index & Dashboard has worked as a powerful tool to bring SDGs clearly and firmly into the policy arena in our States and UTs [13]. Ministry of Statistics and Programme Implementation (MoSPI), Government of India developed a National Indicator Framework (NIF), which is the backbone for facilitating monitoring of SDGs at the national level and provides appropriate direction to the policymakers and the implementing agencies of various schemes and programmes [14].

The main objective of this study is to find out the status of SDG target 6.1, 6.2, 6.4 and 6. towards the achievement of SDG 6 in the 2030 agenda in India (National and State level) and to assess deficiency towards the Achievement of clean Water and sanitation for all in 2030 agenda India (National and State level).

## Materials and methods

The present study is based on seven indicators of SDG 6;

- a: those are % population having improved source of drinking water- SDG 6.1,
- b: % of individual household toilets constructed against target (SBM(G))- SDG 6.2,
- c: % of districts verified to be ODF (SBM(G))- SDG 6.2,
- d: % of school has a separate toilet for boys and girls- SDG 6.2,
- e: % of households having safe disposal of liquid waste- SDG 6.a,
- f: % of blocks/ mandals / taluka having safe groundwater extraction—SDG 6.4, and.
- g: % of blocks/ mandals / taluka over-exploited- SDG 6.4. Data of those indicators are collected from the following secondary sources:

**Table:** 

Types of Data	Sources
Improved sources of drinking water and access to latrine facilities in rural, urban and total	NSS 69th (2012) and 76th (2018) round
Percentage of blocks/ mandals / taluka in safe Groundwater extraction and Percentage of blocks/ mandals/ taluka over-exploited	Central Ground Water Board, Department of Water Resources, River Development and Ganga Rejuvenation Government of India annual report 2018–2019
Household accessibility to the toilet, safe disposal of liquid waste, separate toilets for boys and girls in rural India	National Annual Rural Sanitation Survey (2019–20)
Household toilet coverage (%) and open defecation-free village (%)	Swachh Bharat Mission Gramin Dashboard,2020
Population having improved source of drinking water	Department of Drinking Water and Sanitation, Ministry of Jal Shakti, January 2021

The present study is based on percentage distribution, normalization and arithmetic mean methods. The percentage of groundwater extraction from extractable groundwater resource annually is calculated by the formula: $\left(\frac{\mathrm{total}\;\mathrm{annual}\;\mathrm{groundwater}\;\mathrm{extraction}}{\mathrm{annual}\;\mathrm{extractable}\;\mathrm{groundwater}\;\mathrm{resource}}\times100\right)\%$. And goal score for SDG 6 indicators is calculated by target setting, followed by normalizing the raw data of indicator arithmetic mean of the normalizing value of indicators. The methodology of goal score calculation was developed by the Ministry of Statistics and Programme Implementation (MoSPI) in 2019. The target of those indicators was set by United Nations at the global level. The national target value for indicator a:100%, b:100%, c:100%, d:100%, e:100%, f:100% and g:0%. The next step is normalizing the raw data. It is important to maintain a standard indicator value between 0 and 100. An indicator higher value = lower performance, following formula, was used – the normalized value of an indicator $({N}_{V})=\left(1-\frac{\mathrm{Actual }\;\mathrm{value}\;\mathrm{of}\;\mathrm{an}\;\mathrm{indicator}\;\left(\mathrm i\right)-\mathrm{target}\;\mathrm{value}\;\mathrm{of}\;\mathrm{the}\;\mathrm{indicator}\;(\mathrm i)}{\mathrm{maximum}\;\mathrm{value}\;\mathrm{of}\;\mathrm{the}\;\mathrm{indicator}(i)-\mathrm{Target}\;\mathrm{value}\;\mathrm{of}\;\mathrm{the}\;\mathrm{indicator}\;\left(\mathrm i\right)}\right) \times100$. Normalization does not require for indicators a, b, c, d, e & f because values of that indicator are already in percentage and g have been done using the above formula. The goal score for all indicators of SDG 6 for each state and UTs have been done by the arithmetic mean of normalized value, using the following formula- Goal score of indicator(GSI) = (${\sum }_{i=1}^{Ni}Nv$ and $Av$ × $\frac{1}{\mathrm{Ni}}$). Whereas Ni means = the number of non-null indicators and $Nv$ means the normalized value of the indicator and *Av* means the actual value of the indicator.

## Result and Discussion

**Fig. 1:**
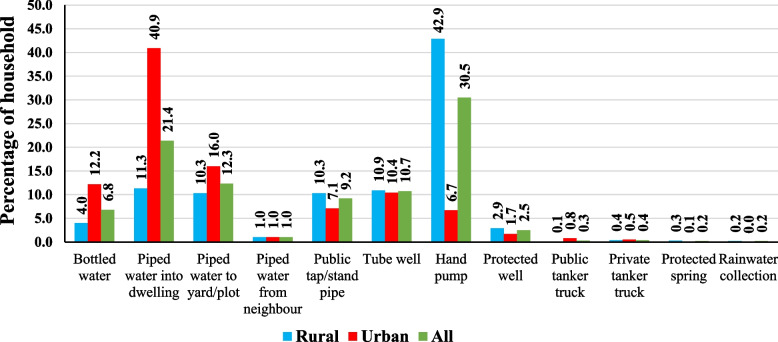
Percentage of households with access to principle sources of safe drinking water in India with resident type, 2018. Source: NSS 76th round (July—December 2018), graph prepared by the author. Notes: 0.0% indicate the least or negligible Percentage of household

Result of households having access to Drinking Water (SDG 6.1) in India (National level and state level) as per National Sample Survey (NSS) data. Figure 1 depicts the sources of safe drinking from households accessing the drinking water throughout the year.

In India 2018, most of household collect safe drinking water from hand pump (30.5%) followed by piped water into dwelling (21.4%), piped water to yard / plot (12.3%), tube well (10.7%), public tap / standpipe (9.2%), bottled water (6.8%), protected well (2.5%), piped water from neighbour (1.0%), private tanker truck (0.4%), public tanker truck (0.3%), protected spring (0.2%) and rainwater collection (0.2%). In urban areas, a higher percentage of households use piped water into the dwelling (40.9%), piped water into yard/plot (16.0%), bottled water (12.2%), public tanker truck (0.8%), private tanker track (0.5%) than a rural area. In rural area higher percentage of household using hand pump (42.9%), tube well (10.9%), public tap / standpipe (10.3%), protected well (2.9%), protected spring (0.3%) and rainwater collection (0.2%) [14].

"Bottled water, piped water into dwelling, piped water to yard/plot, public tap/standpipe, tube well/borehole, protected well, protected spring and rainwater collection are considered as improved sources of drinking water" [15]. As of 2018, 88.7% of households have access to drinking water from principal drinking water sources throughout the year, but 95.5% of household’s access improved drinking water sources in India. In contrast, the urban area has a higher percentage of access to principle (90.9%) and improved (97.4%) drinking water sources throughout the year than the rural area 87.6% and 94.5%, respectively. In India, 1.7% of principle sources and 4.9% improved drinking water sources increased from 2012 to 2018. As of 2018, 11.3% of households have a deficit in case of access principle sources of drinking water, and 4.5% of households have an obligation in case of access to improved sources of drinking water throughout the year for achieving safe and affordable drinking water for all (SDG 6.1) in 2030 agenda. Table 1 showing the percentage of households with access and deficit to drinking water with resident type in India.

**Table 1:** Percentage of households with access & deficit to drinking water with resident type in India

Years	Percentage of households having access to drinking water			Share of household having deficit to reach SDG 6.1 in 2030 agenda		
	Rural	Urban	All	Rural	Urban	All
The principle source of drinking water throughout the year						
2018	87.6	90.9	88.7	12.4	9.1	11.3
2012	85.8	89.6	87.0	14.2	10.4	13.0
Improved principal sources of drinking water throughout the year						
2018	94.5	97.4	95.5	5.5	2.6	4.5
2012	88.5	95.3	90.6	11.5	4.7	9.4

Sources: NSS 76th round (July—December 2018) & 69th round (July—December 2012), table calculated by the author

**Fig. 2:**
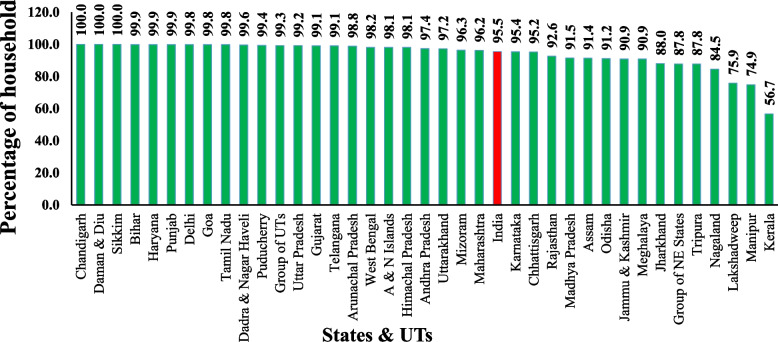
Percentage of households having access to improved sources of drinking water in states & UTs in India, 2018. Source: NSS 76th round (July—December 2018), graph prepared by the author

From Fig. 2, we can say the performance of states and UTs in India towards the Achievement of SDG 6 of target SDG 6.1 by using the percentage of households having access to improved sources of drinking water indicator. As per 2018, SDG 6.1 target achiever ( 100%) states and UTs are Chandigarh, Daman and Diu, Sikkim; Front Runner (65%– 99%) States and UTs are Bihar, Haryana, Punjab, Delhi, Goa, Tamil Nadu, Dadra and Nagar Haveli, Puducherry, Group of UTs, Uttar Pradesh, Gujarat, Telangana, Arunachal Pradesh, West Bengal, Andaman and Nicober Islands, Himachal Pradesh, Andhra Pradesh, Uttarakhand, Mizoram, Maharashtra, Karnataka, Chhattisgarh, Rajasthan, Madhya Pradesh, Assam, Odisha, Jammu and Kashmir, Meghalaya, Jharkhand, Group of NE States, Tripura, Nagaland, Lakshadweep and Manipur; performer state (50%—64%) in Kerala. Kerala has lower access to improved safe drinking water sources. Deficit of performance to achieve SDG 6.1 target based on the above indicator for states and UTs in India are Bihar 0.1%, Haryana 0.1%, Punjab 0.1%, Delhi 0.2%, Goa 0.2%, Tamil Nadu 0.2%, Dadra and Nagar Haveli 0.4%, Puducherry 0.6%, Group of UTs 0.7%, Uttar Pradesh 0.8%, Gujarat 0.9%, Telangana 0.9%, Arunachal Pradesh 1.2%, West Bengal 1.8%, Andaman and Nicober Islands 1.9%, Himachal Pradesh 1.9%, Andhra Pradesh 2.6%, Uttarakhand 2.8%, Mizoram 3.7%, Maharashtra 3.8%, Karnataka 4.6%, Chhattisgarh 4.8%, Rajasthan 7.4%, Madhya Pradesh 8.5%, Assam 8.6%, Odisha 8.8%, Jammu and Kashmir 9.1%, Meghalaya 9.1%, Jharkhand 12%, Tripura 12.2%, Nagaland 15.5%, Lakshadweep 24.1%, Manipur 25.1% and Kerala 43.3%. Although Kerala has a higher socio-economic development performance, Kerala faces a water crisis. "Urbanisation, modernisation, increasing material prosperity, the disintegration of traditional joint family structure, pressure on land, replacing open dug well with bore well, overexploitation of groundwater contribution to the water crisis in Kerala" [16]. "Kerala received 80% less rainfall than normal after a flood. So more dry spells and drops in groundwater levels are one of the reasons for the water crisis." (V P Dineshan). In terms of households having toilet facilities, all northeastern states exceed the national average. However, except with Arunachal Pradesh and Sikkim, all northeastern states are below the national average regarding access to improved drinking water sources.

Similarly, the percentage of villages in Arunachal Pradesh, Assam, Manipur and Meghalaya where the “Village Health and Sanitation Committee” exist is less than the national figure. Efforts should be made to form a "Village Health and Sanitation Committee" in an increasing number of villages. Financial assistance should promote family toilets and provide safe drinking water [17].

Result of households having access to latrine facility (SDG 6.2) in India (National level and state level) as per National Sample Survey (NSS) data.

**Fig. 3:**
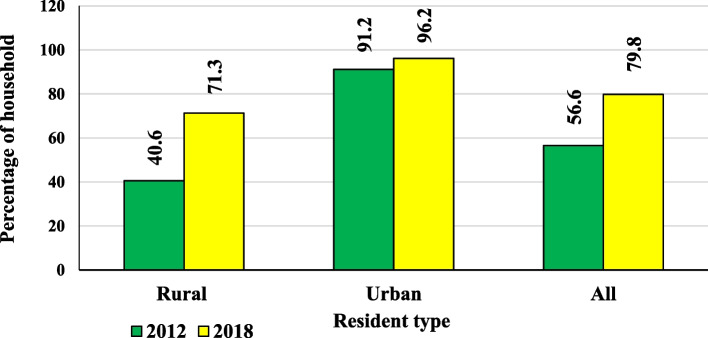
Percentage of households having access to latrine facility with resident type, 2012 & 2018. Sources: NSS 76th round (July—December 2018) & 69th round (July—December 2012), graph prepared by the author

As per 2018, in India, 79.8% of households have access to latrine facilities, whereas urban area has a higher percentage of household having access to latrine facility (96.2%), than rural areas (40.6%) given in the Fig. 3. From 2012 to 2018, India had a 23.2% improvement in accessing latrine facilities, where the urban area has 5%, and the rural area has 30.7% improvement. As of 2018, in India, 20.2% of households have a deficit in accessing latrine facilities towards achieving SDG 6.2 in 2030, whereas in an urban area, it is a low deficit (3.8%) and in rural areas, it is a higher deficit  (28.7%).

As per NSS 76th round, it is seen that in 2018 in India, 2.8% of the population never used a toilet. Although households have latrine facilities, it is higher in rural areas at 3.5% and lowers in an urban area at 1.7%. The various reasons behind not using the toilet are that 2.8% there is no superstructure, 8.2% impure unclear and insufficient water, 3% malfunctioning of the latrine, 0.5% deficiency of latrines, 1.3% lack of safety, 6.3% personal preference, 0.6% cannot bear the charge of the paid latrine, and another reason is 76.9%. It is also observed that the female population uses toilets more than the male population. 74.1% of households washed their hands with water and soap/detergent, and 13.4% washed their hands with water only after defecation [14]. Infrastructure is inadequate in the rural sanitation sector that must be addressed through immediate legislative reforms and government subsidies to develop appropriate and adequate facilities [18].

**Fig. 4:**
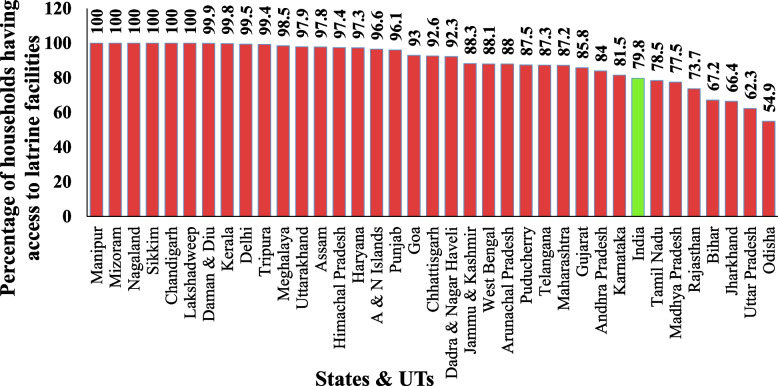
Percentage of households having access to latrine facilities in states & UTs in India, 2018. Source:NSS 76th round (July—December 2018), graph prepared by the author

Figure 4 showing the Percentage of households having access to latrine facilities. A higher percentage of households having access to latrine facilities is found in Manipur, Mizoram, Nagaland, Sikkim, Lakshadweep, etc. A lower percentage of households below the national level are found in Odisha, Uttar Pradesh, Jharkhand, Bihar, Rajasthan, Madhya Pradesh and Tamil Nadu. Inadequacies in rural infrastructure are undoubtedly a significant source of the 'failure.' It has multiple causes, which can be baffling at times. Government-subsidized latrines in rural areas are often inappropriate, especially for women, due to a lack of roofs, doors, walls, buried pits, and adequate spatial dimensions, each of which depends on the convenience of latrine usage and, more crucially, privacy [18]. Performance of states and UTs in India towards the Achievement of SDG 6 of target SDG 6.2 by using the percentage of households having access to latrine facility indicator.

As per 2018, SDG 6.2 target achiever (100%) states and UTs are Manipur, Mizoram, Nagaland, Sikkim, Chandigarh and Lakshadweep; front runner ( 65%– 99%) states and UTs are Daman and Diu, Kerala, Delhi, Tripura, Meghalaya, Uttarakhand, Assam, Himachal Pradesh, Haryana, Andaman and Nicober Islands, Punjab, Goa, Chhattisgarh, Dadra and Nagar Haveli, Jammu and Kashmir, West Bengal, Arunachal Pradesh, Puducherry, Telangana, Maharashtra, Gujarat, Andhra Pradesh, Karnataka, Tamil Nadu, Madhya Pradesh, Rajasthan, Bihar and Jharkhand; performer (50% to 64%) states are Uttar Pradesh and Odisha. As per 2018, deficit of performance towards achievement of SDG 6.2 target in 2030 agenda in States and UTs in India are Daman and Diu 0.1%, Kerala 0.2%, Delhi 0.5%, Tripura 0.6%, Meghalaya 1.5%, Uttarakhand 2.1%, Assam 2.2%, Himachal Pradesh 2.6%, Haryana 2.7%, Andaman and Nicober Islands 3.4%, Punjab 3.9%, Goa 7%, Chhattisgarh 7.4%, Dadra and Nagar Haveli 7.7%, Jammu and Kashmir 11.7%, West Bengal 11.9%, Arunachal Pradesh 12%, Puducherry 12.5%, Telangana 12.7%, Maharashtra 12.8%, Gujarat 14.2%, Andhra Pradesh 16%, Karnataka 18.5%, Tamil Nadu 21.5%, Madhya Pradesh 22.5%, Rajasthan 26.3%, Bihar 32.8%, Jharkhand 33.6%, Uttar Pradesh 37.7% and Odisha 45.1%. The result of the Percentage of blocks/mandals/talisie safe extraction of groundwater (SDG 6.4 and 6.6) in India (National level and state level) as per NSS 76^th^ round data. Infections and illnesses tend to be exacerbated by a lack of latrine facilities. Women and girls are usually disadvantaged due to several socio-cultural and economic factors that deny them equal rights with males. They have distinct physical needs from males, but they also have a greater need for privacy and safety regarding personal cleanliness. Actions such as going long distances in search of a good defecation site and carrying water are a sign of added load, which may be physically unpleasant and hard for women, particularly pregnant women [19].

**Fig. 5:**
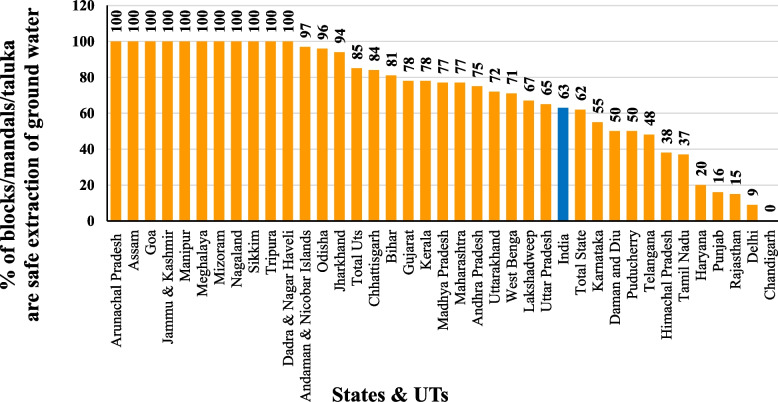
Percentage of blocks/mandals/taluka are safe extraction of groundwater in States & UTs in India,2017. Source: CGWB annual report 2019–2020, graph prepared by the author

Figure 5 showing the Percentage of blocks/mandals/talisie safe extraction of groundwater. As per 2017, the performance of States and UTs in India towards the Achievement of SDG 6.4 and 6.6 in 2030 agenda based on indicator percentage of blocks/mandals/taluka are safe extraction of groundwater (groundwater extraction does not exceed the total annual groundwater recharge, which is below 70% extraction) shows achiever (100%) States and UTs are Arunachal Pradesh, Assam, Goa, Jammu and Kashmir, Manipur, Meghalaya, Mizoram, Nagaland, Sikkim, Tripura, Dadra and Nagar Haveli; Front Runner (65%-99%) are Andaman and Nicobar Islands, Odisha, Jharkhand, Total UT's, Chhattisgarh, Bihar, Gujarat, Kerala, Madhya Pradesh, Maharashtra, Andhra Pradesh, Uttarakhand, West Bengal, Lakshadweep, Uttar Pradesh; performer (50%-64%) are India, Karnataka, Daman and Diu, Puducherry; aspirant (0%-49%) are Telangana, Himachal Pradesh, Tamil Nadu, Haryana, Punjab, Rajasthan, Delhi and Chandigarh. InIndia 63% blocks/mandals/taluka are safe extraction of groundwater.

**Fig. 6:**
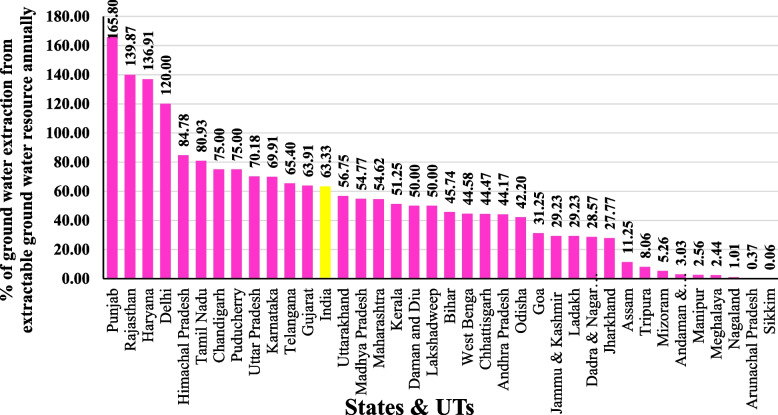
Percentage of groundwater extraction from extractable groundwater resource annually in States & UTs in India,2017. Source: CGWB annual report 2019–2020, graph prepared by the author

Result of the percentage of groundwater extraction (SDG 6.4) in India (National level and state level) as per 2017:

As per the "National Compilation on Dynamic Ground Water Resources of India (2017)" report by the CGWB, groundwater extraction below 70 per cent is considered a Safe extraction. Over extraction of groundwater annually (groundwater extraction exceed extractable groundwater annually) is found in Punjab (165.80%), Rajasthan (139.87%), Haryana (136.91%) and Delhi (120.00%); safe groundwater extraction is found in Karnataka, Telangana, Gujarat, India, Uttarakhand, Madhya Pradesh, Maharashtra, Kerala, Daman and Diu, Lakshadweep, Bihar, West Bengal, Chhattisgarh, Andhra Pradesh, Odisha, Goa, Jammu and Kashmir, Ladakh, Dadra and Nagar Haveli, Jharkhand, Assam, Tripura, Mizoram, Andaman and Nicobar Islands, Manipur, Meghalaya, Nagaland, Arunachal Pradesh and Sikkim. In India, 63.33% of groundwater is extracted annually as per 2017. The States and UTs with safe groundwater extraction achieve the SDG 6.4 target based on the indicator – the annual percentage of groundwater extraction from extractable groundwater resources. Figure 6 showing the Percentage of groundwater extraction from extractable groundwater resource annually in States and UTs.

"In India as per 2017 Total Annual Groundwater Recharge is 431.86 billion cubic meters (bcm) out of which Annual Extractable Ground Water Resource is 392.7 bcm and Current Annual Ground Water Extraction is 248. 7 bcm" (CGWB annual report 2019–2020).

### Result of the overall performance of SDG 6 in India (National level and state level) 2019 – 2021.

Table 2 shows the achievements towards SDG 6 of all States and UTs. Overall goal score of the indicator—Percentage of the rural population having improved source of drinking water (SDG 6.1), percentage of individual household toilets constructed against target (SBM(G)) (SDG 6.2), percentage of districts verified to be ODF (SBM(G)) (SDG 6.2), the school has a separate toilet for boys and girl (%) ( SDG 6.2), percentage of Household Safe Disposal of Liquid waste (SDG 6.a), percentage of blocks/ mandals/ taluka having safe groundwater extraction (SDG 6.4) and percentage of blocks/ mandals/ taluka over-exploited (6.4) reveal that states and UTs belonging in achiever stage are Chandigarh, Dadra & Nagar Haveli, Ladakh, Lakshadweep, Sikkim and Goa. The states and UTs belonging to front runner stage (66–99%) are Mizoram, Andaman & Nicobar Islands, Jharkhand, Odisha, Kerala, Gujarat, Chhattisgarh, Jammu & Kashmir, Meghalaya, Arunachal Pradesh, Maharashtra, Uttarakhand, Assam, West Bengal, Nagaland, Tripura, Bihar, Andhra Pradesh, Madhya Pradesh, Uttar Pradesh, Daman and Diu, Puducherry, Telangana, Karnataka, Manipur, Tamil Nadu, Himachal Pradesh, Haryana, Rajasthan and Punjab. Delhi is the only Union Territory belonging to the aspirant stage.

**Table 2:**
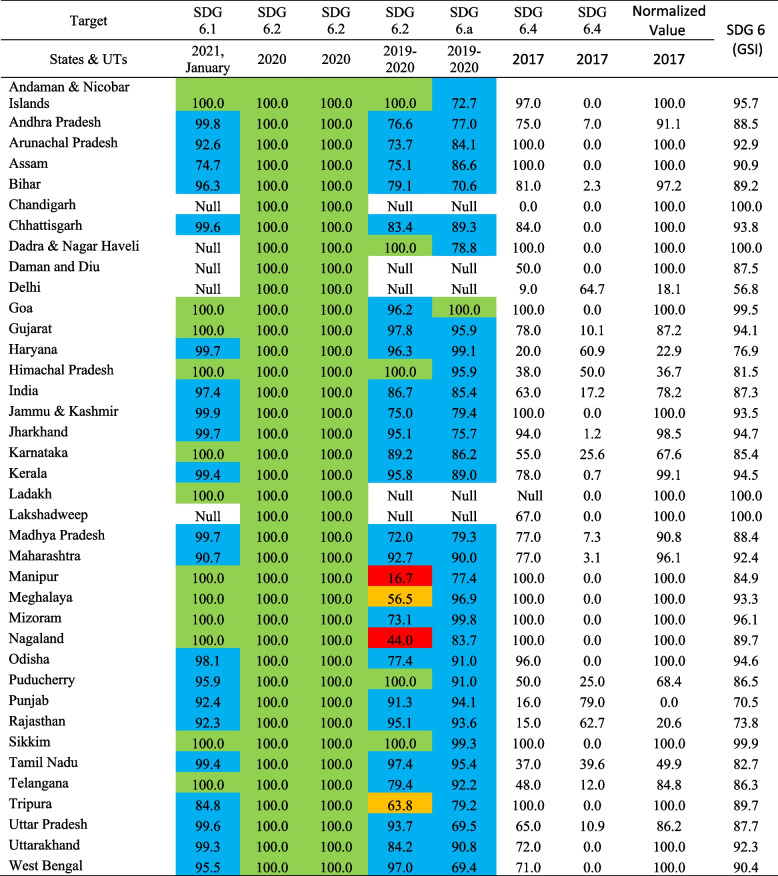
Overall performance of Rural India towards Achievement of SDG 6, 2019-2021

Sources: Department of Drinking Water and Sanitation, Ministry of Jal Shakti, January 2021; Swachh Bharat Mission Gramin Dashboard,2020; NARSS round 3, 2019–2020; table computed by author. Notes: Achiever (100%) Front Runner (65%-99%) Performer (50%-64%) Aspirant (0%-49%)

*note

SDG 6.1Population having improved source of drinking water

SDG 6.2Percentage of individual household toilets constructed against target (SBM(G))

SDG 6.2Percentage of districts verified to be ODF (SBM(G))

SDG 6.2The school has a separate toilet for boys & girls (%)

SDG 6. a% of Household Safe Disposal of Liquid waste

SDG 6.4% of blocks/ mandals / taluka having safe groundwater extraction

SDG 6.4% of blocks/ mandals / taluka over-exploited

SDG 6Goal score of the indicator (GSI)

As per January 2021, the performance of States and UTs in Rural towards Achievement of SDG 6.1 based on indicator Percentage of the rural population having improved source of drinking water shows achiever States and UTs are; Ladakh, Sikkim, Goa, Mizoram, Andaman & Nicobar Islands, Gujarat, Meghalaya, Nagaland, Telangana, Karnataka, Manipur and Himachal Pradesh; front runner are Jammu & Kashmir, Andhra Pradesh, Jharkhand, Madhya Pradesh, Haryana, Chhattisgarh, Uttar Pradesh, Kerala, Tamil Nadu, Uttarakhand, Odisha, Bihar, Puducherry, West Bengal, Arunachal Pradesh, Punjab, Rajasthan, Maharashtra, Tripura and Assam.

**Fig. 7:**
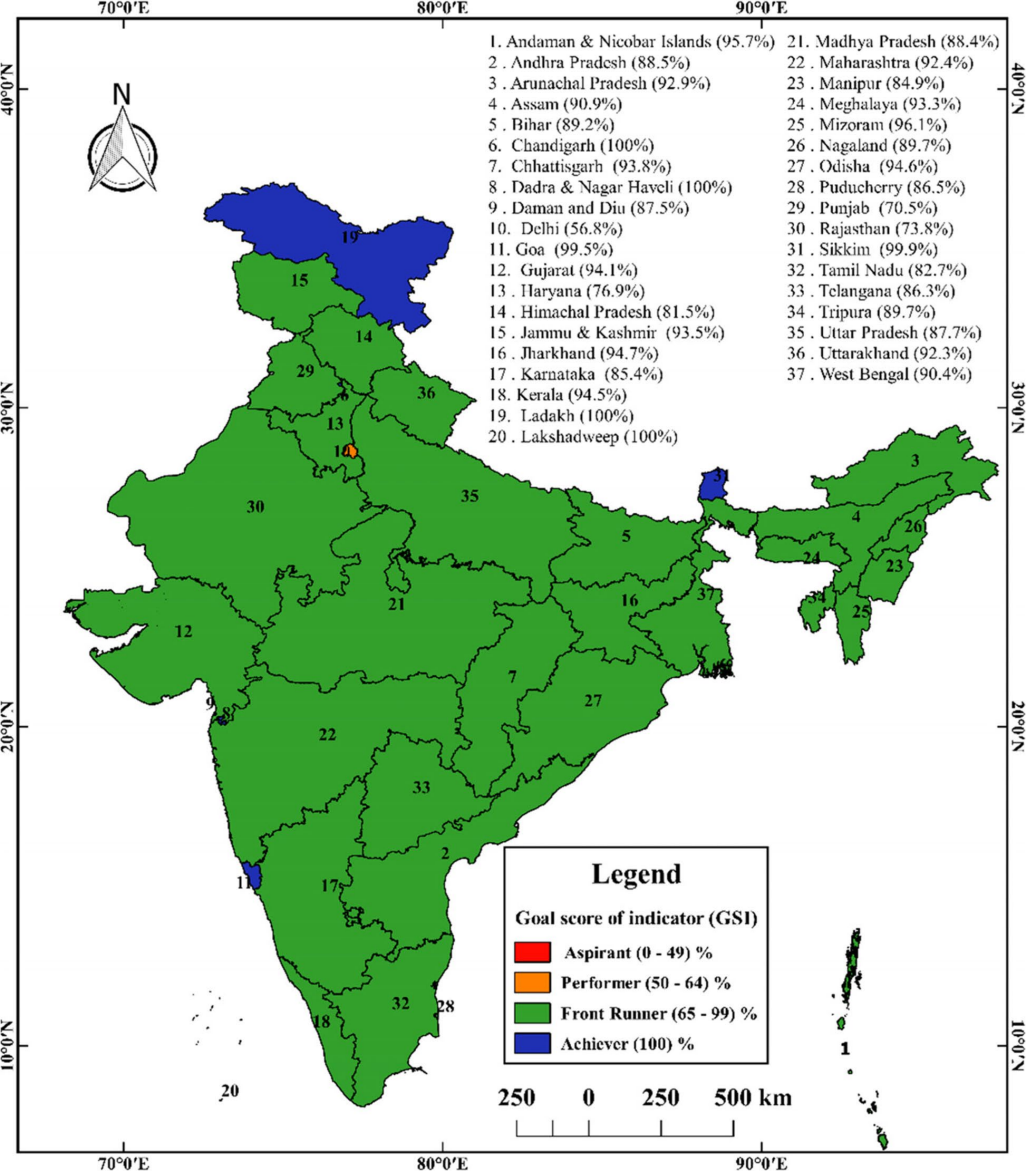
Overall performance of different indicators of SDG 6 (Goal score of the indicator). Sources: Department of Drinking Water and Sanitation, Ministry of Jal Shakti, January 2021; Swachh Bharat Mission Gramin Dashboard,2020; NARSS round 3, 2019–2020; map prepared by the author

From Fig. 7, we can see that most of the states and union territories belong to the green colour shade. That means all these states and union territories are in the Front Runner (65–99%) stage as per the Goal Score Indicator (GSI). Andaman & Nicobar Islands, Chandigarh, Dadra & Nagar Haveli, Ladakh, Lakshadweep, Sikkim and Goa are all states and Union Territories observing blue colour shade, indicating that all these states and union territories have reached the achiever stage as per the Goal Score Indicator (GSI). Delhi is the only union territory where orange colour is observed, indicating that the union territory is still at the performer (50–64%) stage.

**Fig. 8:**
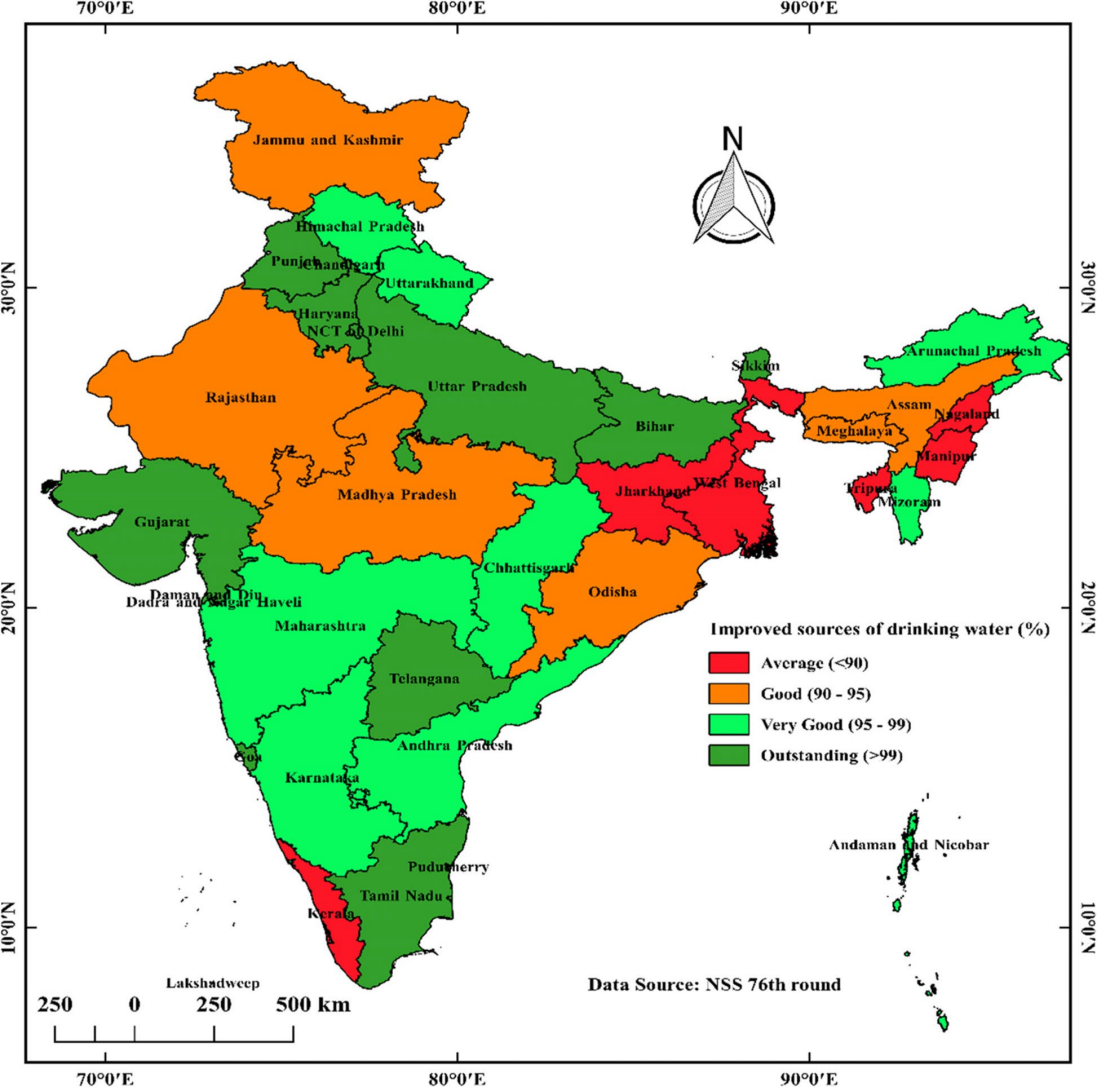
Spatial distribution of households having access to improved sources of drinking water (%) in states & UTs in India, 2018. Source: NSS 76th round (July—December 2018), map prepared by the author

**Fig. 9:**
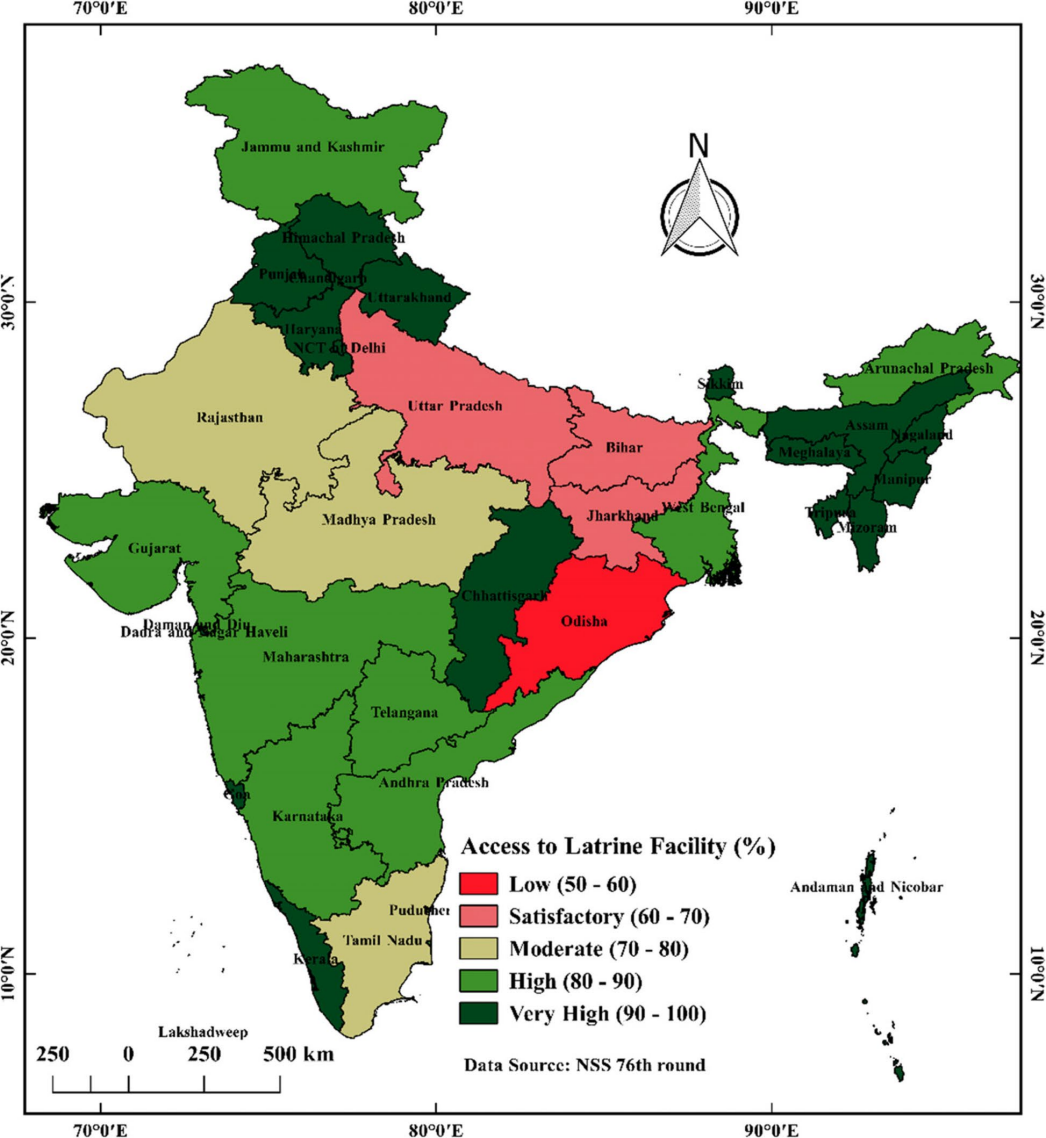
Spatial distribution of households having access to latrine facility (%) in states & UTs in India, 2018. Source: NSS 76th round (July—December 2018), map prepared by the author

Figure 8 shows the spatial distribution of households having access to improved sources of drinking water and Fig. 9 shows the spatial distribution of households having access to latrine facilities in States and UTs in India.

From Fig. 8, light green indicates states and union territories with 95–99% coverage of improved drinking water sources. Moreover, deep green indicates those states and union territories with more than 99% coverage of improved drinking water sources. The red colour indicates below 90% coverage of improved drinking water sources. Furthermore, orange indicates those states and union territories with 90–95% coverage of improved drinking water sources. All South Indian states except Kerala fall into more than 95% coverage of improved drinking water sources. Almost all States and Union Territories above and near the Tropic of Cancer have < 95% coverage of Improved Sources of Drinking Water except Chhattisgarh and Gujarat. Almost all states of North India except Jammu and Kashmir have more than 95% coverage of improved drinking water sources.

From Fig. 9, light green indicates states and union territories with 80–90% coverage of access to latrine facilities. Moreover, deep green indicates those states and union territories with 90–100% coverage of access to latrine facilities. The red indicates below 50–60% coverage of access to latrine facilities. Furthermore, pink indicates those states and union territories with 60–70% coverage of access to latrine facilities. Whitish Grey indicates states and union territories with 70–80% coverage of access to latrine facilities. Delhi, Uttar Pradesh, Bihar, Jharkhand, and Odisha fall into less than 70% coverage of access to latrine facilities. Rajasthan, Madhya Pradesh and Tamil Nadu are found to have 70–80% coverage of access to latrine facilities. The rest of the states and union territories have found more than 80% coverage of access to latrine facilities.As per NSS data in 2018, 30.5% of households collect safe drinking water from the hand pump; in the case of urban areas 40.9% of households use piped water into the dwelling; and in rural areas 42.9% of households use the hand pump. 88.7% of households have access to a principle source of drinking water, and 95.5% use improved drinking water sources throughout the year. 100% of households having access to improved sources of drinking water (SDG 6.1 target achiever) in Chandigarh, Daman and Diu, Sikkim and Kerala has the lowest percentage 56.7%. In India, 79.8% of households have access to latrine facilities, whereas urban area has a higher percentage of household having access to latrine facility 96.2%, than rural areas (40.6%). The female population are more using toilets than the male population. 100% of households have access to latrine facilities (SDG 6.2 target achiever) in Manipur, Mizoram, Nagaland, Sikkim, Chandigarh, Lakshadweep; and the lowest found in Odisha 54.9%. Safe groundwater extraction from extractable groundwater resources annually (SDG 6.4 target achiever) in States and UTs in India, 2017 are found in Karnataka, Telangana, Gujarat, India, Uttarakhand, Madhya Pradesh, Maharashtra, Kerala, Daman and Diu, Lakshadweep, Bihar, West Bengal, Chhattisgarh, Andhra Pradesh, Odisha, Goa, Jammu & Kashmir, Ladakh, Dadra and Nagar Haveli, Jharkhand, Assam, Tripura, Mizoram, Andaman and Nicobar Islands, Manipur, Meghalaya, Nagaland, Arunachal Pradesh and Sikkim. In India, 63.33% of groundwater is extracted annually as per 2017. As of 2020, all the States and UTs in Rural India 100% individual household toilets constructed against target (SBM(G)) and 100% districts verified to be ODF (SBM(G)) (SDG 6.2 target achiever). As per January 2021, 100% rural population has improved source of drinking water (SDG 6.1 target achiever) in Ladakh, Sikkim, Goa, Himachal Pradesh, Gujarat, Karnataka, Mizoram, Andaman and Nicobar Islands, Telangana, Meghalaya, Nagaland and Manipur. As per 2019–2020, 100% school having a separate toilet for boys and girl (SDG 6.2 target achiever) in Dadra and Nagar Haveli, Sikkim, Himachal Pradesh, Andaman and Nicobar Islands and Puducherry. Goa achieves 100% safe disposal of liquid waste. Overall goal score expresses all the states belong to front runner stage (65% to 99%). Based on SDG 6.1 and SDG 6.2, it is observed that in Rural India achiever (100%) state is Sikkim, Himachal Pradesh, Andaman and Nicobar Islands in 2019–2021.Since the population is increasing, the number of sustainable water resources is not. Future population expansion will likely result in further reduced renewable water available per capita. Most changes in India's overall and rural regions, moderate changes in the world's overall and rural areas, and very little change in both India's and the world's urban areas have been seen in terms of access to essential drinking water services [20]. The top eight states are Gujarat, Jammu & Kashmir, Madhya Pradesh, Andhra Pradesh, Odisha, Maharashtra, Karnataka, and Telangana; the bottom eight are Delhi, Uttarakhand, Haryana, Uttar Pradesh, Bihar, Kerala, and West Bengal. Due to their location in the Ganges basin, most of the eight lowest performing states have abundant water resources, in contrast to the higher performing states, which are comparatively water scarce. Severe droughts have recently affected Gujarat, Maharashtra, Madhya Pradesh, Andhra Pradesh, Karnataka and Telangana. From an endowment standpoint, this focuses the attention of water concerns in India toward improved management and control of water resources. The top five states in terms of performance are Goa, Delhi, Kerala, Gujarat, and Telangana, whereas the worst five are Chhattisgarh, Bihar, Odisha, Andhra Pradesh, and Jharkhand. In Jharkhand, Bihar, and Uttar Pradesh, childhood malnutrition and stunting have increased due to poor sanitation services. Individually, these indices point to significant disparities in access to sanitary facilities and clean water throughout the states. Few states have been able to implement comprehensive planning to meet the key objectives [21, 22].

The WHO/UNICEF, joint monitoring program estimated in 2012 that 60% of the world's open defecation occurs in India. While this trend is declining rapidly in other countries, it continues stubbornly in India. According to the 2011 Census of India data, about 90% of rural people in India defecate in the open. Social context always plays a vital role in countries like India, where households with higher income and better education are more likely to use latrines and toilets. Previous research has shown that Muslims are 25% less likely to defecate in the open than Hindus. Although Hindus have 6% more per capita consumption than non-Hindus, Hindus are less likely to use latrines [23].

Open defecation at the individual level is a more accurate reflection of the disease environment than latrine ownership at the household level. It is particularly true in rural India, where earlier research has shown that many residents of homes with latrines do not use their latrines. The literature indicates that the Indian government's policy of subsidizing pit latrines has not achieved large-scale behaviour change and may still represent a misguided focus. This policy has continued mainly under the current Swachh Bharat Mission (2014–present). Despite the evidence, understanding latrine demand is critical to understanding latrine uptake [24, 25]. Sanitation practices and social norms receive minimal consideration in sanitation programmes. Sanitation policy would probably be more effective if it addressed the underlying social environment in which judgments about where to defecate and what kind of latrine is socially acceptable since even the well-educated and wealthiest households adopt latrines at such a slow pace [26].

## Conclusion

After lunch of Swachh Bharat Mission and other programmes related to sanitation and drinking water, sanitation coverage and accessibility of drinking water rise which has reinforcement substantially in accelerating the Achievement of Sustainable Development goal 6. States and UTs having the lower status of sanitation, drinking water, groundwater and hygiene need to improve those condition by increasing availability, accessibility and affordability of the WASH facility. Localisation or bottom-up approach by giving responsibility to rural and urban local body enforced Achievement of SDG 6. Total water withdrawal per capita was 576.96 m^3^ in 1975, which was 602.3 m^3^ in 2010. Total water withdrawal has increased by about 3.07% in these few decades. From 1962 to 2014, 64.29% per capita of total internal renewable water resources decreased. From 1979 to 2011, 18.4% increase in water stress. To fulfil essential human needs and attain sustainable development aims, central and local governments must collaborate. These initiatives and actions for recyclable and reusable, sufficient, and treated water, as well as enhancing sanitation and hygiene infrastructure, are linked to creating opportunities that improve economic sustainability. Additionally, establishing sanitation, hygiene and drinking water infrastructure in households grants social dignity, which can assist in social sustainability.

Those States and Union Territories that have not achieved the goal of 100% overall SDG-6 should fulfil the goals through a specific regional development approach. If successful locally, it will help the country's overall progress on a large scale. India and other underdeveloped and developing countries need to fulfil the goals of SDG-6. If successful in achieving the target, it will accelerate overall health improvement and help reduce regional disparities. Developed countries need to help developing and underdeveloped countries. Finally, the various organizations of the United Nations should try to solve the problems at the local level through each country-specific regional approach that will accelerate the overall achievement.

To prevent and reduce acute and chronic illness death and sustain a healthy life, we need to increase awareness and facilities to access safe and adequate drinking water, sanitation and hygiene. For raising awareness, different days are celebrated on 22 March as World Water Day for Water, 19 November as World Toilet Day for sanitation and 15 October as Global Handwashing Day for hygiene. Still, we need to maintain safe drinking water, sanitation and hygiene all day. 

## Data Availability

The study is based on secondary data analysis. No data was collected for this study. The datasets generated and/or analysed during the current study are available in the NSS (Download Reports | Ministry of Statistics and Program Implementation | Government Of India), Central ground water control board (Department of Drinking Water and Sanitation, GOI (jalshakti-ddws.gov.in)), NARSS (Department of Drinking Water and Sanitation, GOI (jalshakti-ddws.gov.in)) NITI Aayog (Reports on SDG | NITI Aayog) repository.

## Abbreviations

NSS: National Sample Survey
SDGs: Sustainable Development Goals
